# Predictive Factors Associated with Successful Response to Percutaneous Adhesiolysis in Chronic Lumbar Radicular Pain

**DOI:** 10.3390/jcm12196337

**Published:** 2023-10-03

**Authors:** Halil Cihan Kose, Omer Taylan Akkaya

**Affiliations:** 1 Department of Pain Medicine, Kocaeli City Hospital, 41060 Kocaeli, Turkey; 2 Department of Pain Medicine, Health Science University Etlik City Hospital, 06120 Ankara, Turkey; taylanakkaya.md@gmail.com

**Keywords:** chronic pain, percutaneous adhesiolysis, lumbosacral spinal stenosis, visual analogue scale, chronic radicular pain, foraminal stenosis, depression, treatment outcome, interventional, outcome assessment

## Abstract

Percutaneous adhesiolysis (PEA) is of interest in the treatment of lumbar radicular pain. This study aimed to assess the effectiveness of PEA in patients with chronic lumbar radicular pain refractory to epidural steroid injections and to determine predictive factors, including demographic, clinical, and procedural data, to provide superior treatment efficacy. One hundred and ninety-three patients were reviewed. Successful treatment outcome was described as a 50% reduction in the visual analog scale score. Among the 193 patients, 109 (56.2%) exhibited a positive treatment response at 12 months. In multivariate logistic regression analysis, no depression (OR, 3.105; 95% CI, 1.127–8.547; *p* = 0.028), no spondylolisthesis (OR, 2.976; 95% CI, 1.246–7.092; *p* = 0.014), no previous lumbar surgery (OR, 2.242; 95% CI, 1.067–4.716; *p* = 0.033), mild foraminal stenosis (OR, 3.460; 95% CI, 1.436–8.333; *p* = 0.006), no opioid use (OR, 1.782; 95% CI, 0.854–3.717; *p* = 0.123), and baseline pain scores (OR, 0.787; 95% CI, 0.583–1.064; *p* = 0.120) were the predictive factors significantly associated with unsuccessful treatment outcome. PEA is a useful treatment option for patients with chronic lumbar radicular pain refractory to epidural steroid injections. A history of lumbar surgery, spondylolisthesis, depression, and severe foraminal stenosis could be associated with a poor prognosis.

## 1. Introduction

Lumbar radicular pain, defined as low back pain accompanied by pain in the lower extremities is a frequent symptom in populations worldwide [1]. The components that constitute the lumbar spine, such as vertebrae, facet joints, intervertebral discs, and neurovascular elements, are susceptible to an array of stressor factors [2]. These structural elements, either independently or in combination, have the potential to cause lumbar radicular pain. Chronic lumbar radicular pain is most frequently correlated with conditions such as lumbar spinal stenosis, herniated intervertebral discs, and failed back surgery syndrome [3,4,5].

The management of lumbar radicular pain consists of a stepwise care approach due to the complexity of the contributors to both the pain and physical disability, such as psychosocial and biophysical factors [6]. Treatment options are considered by many clinical guidelines, and recommendations may vary. Due to ongoing concerns about the side effects of opioids and suboptimal outcomes with other pharmacological agents, initial non-pharmacological options, including exercise, physical therapy, and psychological programs, are suggested [7]. Generally, once conservative approaches have failed, a consideration of a wide range of surgical interventions and nerve root injections is recommended [8]. Epidural steroid injections are a widely performed treatment modality to fill the gap between conservative treatments providing limited pain relief and surgical procedures with an estimated incidence of about 10–40% persistent pain [9]. The effectiveness of epidural steroid injections depends on the injectate reaching the target lesion; however, the evidence of treatment success is moderate in patients with spinal stenosis, and the disadvantage is that a perineural fibrosis or an epidural adhesion may lead to failure of the procedure [10,11,12].

Percutaneous epidural adhesiolysis (PEA) is a minimally invasive treatment option in which a catheter is located into the ventral epidural space or around the nerve root. PEA is associated with long-term benefits for symptom control and reducing the long-term risk of surgery in patients with lumbar radicular pain unresponsive to conservative treatment modalities [13,14]. The goal of the PEA procedure is to improve the delivery of pharmacological agents to the target area, thereby overcoming the limitations of epidural injections such as perineural fibrosis or adhesions. Over the past decade, there has been growing evidence that PEA has an important role in chronic radicular pain, with both optimism and concerns among physicians [15,16,17,18,19]. However, little is known about demographic, clinical, and radiological factors that may affect treatment outcomes. Although PEA is considered a safe procedure, it may result in rare but serious adverse events, including dural puncture, epidural abscess, and a torn catheter [20]. Therefore, there is a need to elucidate the selection criteria for PEA procedures, aiming to enhance treatment outcomes, minimize complications, and decrease unnecessary interventions.

This study includes a retrospective analysis of treatment outcomes in a medical center over a 5-year period. The objectives of this trial were to investigate the effectiveness of the PEA procedure in the treatment of chronic lumbar radicular pain among patients for whom epidural injections were not successful and to determine the demographic, clinical, and technical predictors of the outcome of PEA.

## 2. Materials and Methods

Institutional review board approval (Ethics Committee of the Diskapi Yildirim Beyazit Training and Research Hospital 127/22) was obtained. This study was registered at ClinicalTrials.org PRS under Registration No. NCT05235308. This manuscript adheres to the applicable STROBE checklists for observational studies.

### 2.1. Subjects

Patients with a primary complaint of chronic lumbar radicular pain treated with PEA procedures in Diskapi Yildirim Beyazit Training and Research Hospital, Turkey, between January 2016 and January 2021 were included. Patients had undergone PEA if they had lumbar radicular pain lasting more than 3 months, which was decreased by less than 50% by 4 weeks after conservative therapy including physical therapy and oral analgesics and at least two tranforaminal or interlaminar epidural injections administered over 2 months. Pathologies as the source of persistent lumbar radicular pain for at least 6 weeks were confirmed with the MRI findings. All the included patients were age ≥18; refractory to standard treatments, including physical therapy, oral medications, and caudal or transforaminal epidural injections. Our electronic databases were searched using the code ‘551120′ (‘lumbar-caudal percutan adhesiolysis’). Since the patients included in this study were refractory to conventional treatment modalities, including oral medications, physical therapy, and epidural or transforaminal injections, they did not receive additional interventions or physical therapy during the study period. No specific co-interventions were offered. Patients were excluded in cases where adequate follow-up or documentation were missing. Data extraction was hindered under several potential scenarios: patients who missed appointments, those with whom communication was not feasible, or patients who may express dissatisfaction with their treatment and consequently refrain from clinic visits.

### 2.2. Interventions

All the procedures were performed under fluoroscopy in a sterile operating room with standard monitoring. Prior to the initiation of the procedure, the patient’s intravenous access was appropriately established, and antibiotic pretreatment was administered. The patient’s vital signs were meticulously monitored throughout the entire duration of the procedure. The patient was placed in a prone position with a pillow under the abdomen to minimize lumbar lordosis. After local anesthetic infiltration, the 16 G Tuohy needle was inserted through the sacrococcygeal ligament and placed into the epidural space. An epidurogram with 2–5 cc of contrast was obtained to confirm epidural placement of the needle and to avoid intravascular needle placement. Then, a Racz epidural catheter (Epimed, Farmers Branch, TX, USA) was inserted through the epidural needle towards the target area. Approximately 5 cc of radiopaque contrast was injected to identify the filling defects and position of the catheter tip at the anterior epidural space of the target site of pathology. After adhesiolysis was carried out with 1500 units of hyaluronidase, a mixture of 10 cc 0.025% bupivacaine and 4 mg dexamethasone was divided and separately injected into each target. The catheter was removed slowly, and a sterile dressing was applied to the injection site. The patient was positioned in the supine position and transferred to the recovery room, where they were closely monitored for any possible complications or side effects. Vital signs were continuously checked throughout the recovery period in the post-procedure recovery unit. Potential complications during the procedure, such as dura matter puncture or subdural injection, were recorded. In the presence of any suspected neurological complications, the patient underwent serial neurological examinations, and discharge from the hospital was considered only upon the consistent attainment of normal results in these assessments.

### 2.3. Outcome Data and Follow-Up Period

Treatment success was described as achieving at least 50% pain relief compared to the baseline for 12 months, which was defined in previous pain research studies [21,22]. If a patient experienced a positive treatment outcome either at the 1-month or 6-month follow-up and, although there was no further contact during the 12-month follow-up, later returned to our pain clinic and reported ≥ 50% pain relief for 12 months, this situation was also considered a positive outcome. However, patients who had a positive outcome at the 1- or 6-month follow-up but did not maintain any contact afterward were excluded from the final analysis. Patients who received spinal cord stimulators or were transferred to the department of surgery during the study process were also defined as non-responders. Patients with increased oral opioid doses were also classified as non-responders. Throughout the trial, Visual Analog Scale (VAS) scores were collected during clinic visits or via telephone calls at various time points: baseline, 1, 6, and 12 months after the procedure. All patients who participated in the study were categorized based on their treatment response: either as responders with a positive treatment outcome or as non-responders with a negative treatment outcome once the data collection was completed.

After an exhaustive review of prior research concerning the impacts of PEA procedures on treatment effects and subsequent deliberations among the authors of this present study, a comprehensive set of variables was formulated. These variables encompass a wide spectrum of demographic, clinical, and technical clinical attributes. Demographic and clinical variables for logistic regression analysis were age, sex, duration of pain, baseline VAS score, smoking status (former, current, and never), grade of central and foraminal stenosis, employment status (employed or retired-unemployed), depression, obesity (body mass index ≥ 30), history of lumbar surgery, spondylolisthesis, and opioid use. Intervention characteristics included target levels, target sites, and the number of target sites. We conducted a retrospective analysis of the patient’s electronic medical records and image archive system to gather variables. The degree of foraminal and spinal canal stenosis was assessed using established classification standards [23,24]. In cases involving multiple levels of central or foraminal stenosis, the level with the most pronounced stenosis was chosen.

### 2.4. Statistical Analysis

The SPSS version 23.0 statistics program was used. (IBM Corporation, Armonk, NY, USA). We conducted a post hoc power analysis to confirm the adequacy of the sample size for assessing the predictive factor in determining the success of the PEA procedure. Patients in the trial were categorized as responders or non-responders based on their treatment success. Descriptive statistics were summarized by means and standard deviations for continuous outcomes and frequencies (%) for categorical outcomes. We conducted univariate logistic regression analyses utilizing demographic and clinical factors of the patients as potential predictors to quantify the results of treatment success. Thirteen demographic and baseline clinical characteristics and three procedural characteristics were used to perform univariate regression analysis. Multivariate logistic regression analysis was performed with characteristics which were showing a trend towards statistical significance (*p* < 0.200) in univariate analysis. In the univariate analysis, several factors including depression, opioid usage, baseline pain intensity, stenosis severity, spondylolisthesis presence, and history of prior lumbar spinal surgery were identified as the most influential variables. These factors were then incorporated into a multivariate model aimed at predicting the likelihood of procedural success for the PEA procedure. Odds ratio (OR) with 95% confidence interval (CI) was calculated. *p* < 0.05 was considered statistically significant.

## 3. Results

**Figure 1:**
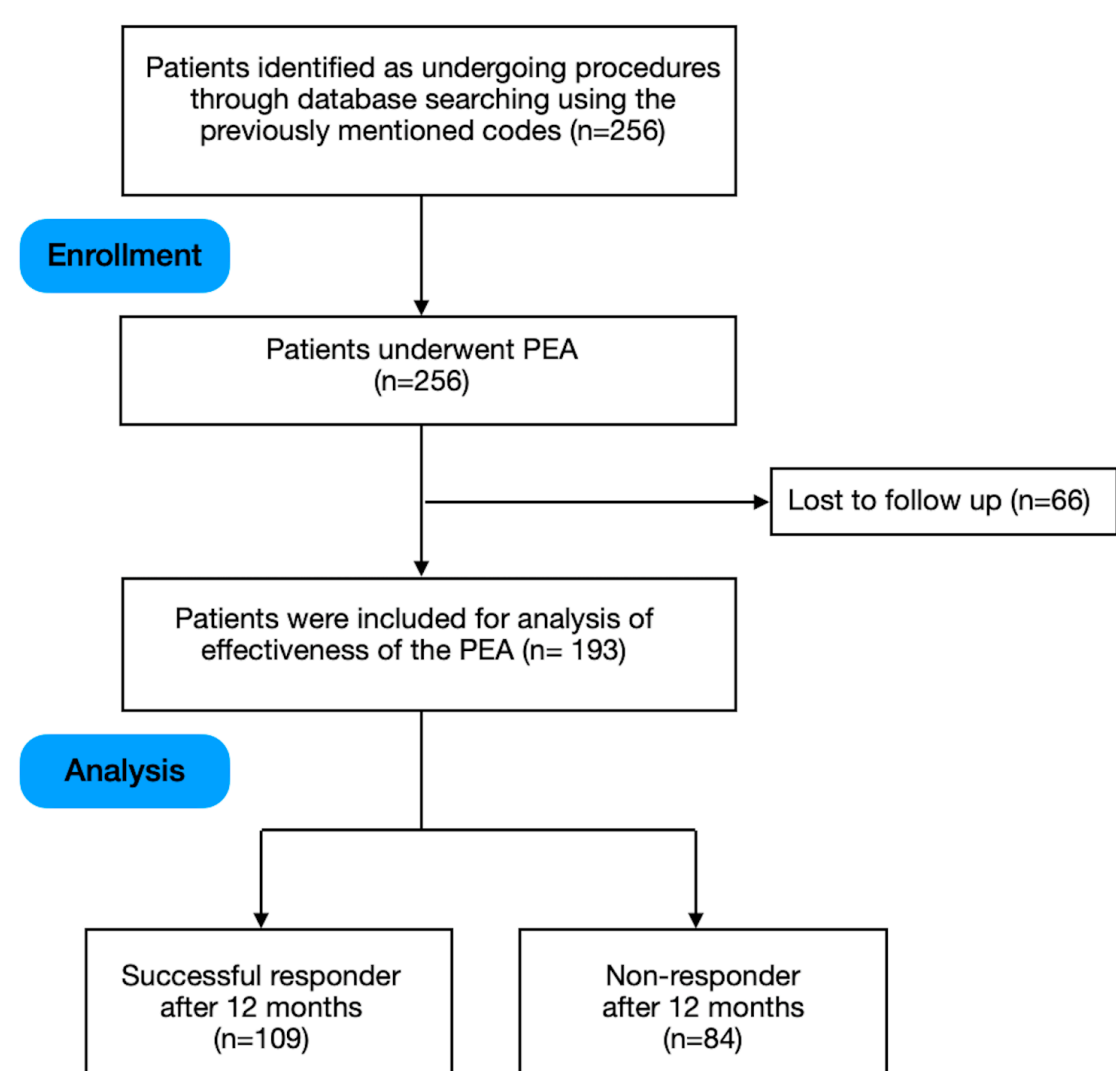
Flowchart of the study.

Using the aforementioned search codes, 259 cases were found to have undergone PEA. Among these, 66 individuals were not included in the research because there were insufficient records. As a result, 193 patients were included in the analysis (Figure 1). In this cohort study, 56.2% (*n* = 109) of the patients achieved a successful treatment outcome at 12 months. In the cohort of non-responder patients, 14 patients had undergone spinal cord stimulation, and 7 patients within this group had undergone surgical interventions. Furthermore, opioid doses were escalated for 30 of the patients as part of their treatment regimen. The study participants’ demographic and baseline clinical characteristics were presented in the following manner: The mean age of the patients was 61.9 ± 10.4. The proportion of females and males was 50.2% and 49.8%, respectively. The average baseline pain score was 7.06 ± 1.3, and the average duration of pain was 10.1 ± 3.72 (months). Of these patients, 30% (*n* = 58) had prior lumbar surgery, 37.3% (*n* = 72) were receiving opioids, 20.2% (*n* = 39) had depression, 33.7% (*n* = 65) were obese, and 23.3% (*n* = 45) had a history of spondylolisthesis (Table 1). The patients’ demographic and clinical characteristics associated with the treatment outcome are demonstrated in Table 1. Post hoc power analysis showed that the effect size for this analysis was f^2^ = 0.67 and the statistical power was 1. The required sample size was 114 to achieve 90% power for a multiple regression on independent variables with α error = 0.05. Considering that 193 patients were enrolled in this study, the post hoc power analysis showed that our analysis met the requirements of sample size calculation.

No significant complications, such as motor weakness, paralysis, hematoma, catheter shearing, or subdural or subarachnoid injections, were observed during the procedure. While some patients did report temporary discomfort and pain at the injection site immediately following the intervention, they all experienced improvement within a few days and did not affect the follow-up period. There were no instances necessitating further medical intervention. Seven patients did encounter a temporary sensory deficit, characterized by radicular numbness in the leg. None of these patients with complications exhibited persistent neurological abnormalities, and all were discharged after a brief period of bed rest to ensure their complete recovery.

### 3.1. Univariate Logistic Regression Analysis

Univariate logistic regression analysis showed that history of spondylolisthesis, history of lumbar surgery, and severe foraminal stenosis were significantly associated with a negative response to PEA using a Racz catheter at 12 months. Factors including no depression (OR: 2.75, 95% CI: 0.95 to 8.33, *p* = 0.062), moderate foraminal stenosis (OR: 2.182, 95% CI: 0.90 to 5.55, *p* = 0.085), no opioid use (OR: 2.09, 95% CI: 0.961 to 4.76, *p* = 0.064), and higher baseline pain scores (OR: 0.626, 95% CI: 0.59 to 1.146, *p* = 0.127) showed a slight trend towards statistical significance (*p* < 0.200). There were no statistically significant differences in factors such as age, sex, symptom duration, obesity, employment, degree of central stenosis, smoking status, and procedural characteristics (Table 2 and Table 3). 

### 3.2. Multivariate Logistic Regression Analysis

Multivariate logistic regression analysis included only those regressors (with a significance level of *p* < 0.200) that exhibited a notable tendency toward statistical significance in the initial univariate analysis. The findings indicate that the multivariate model exhibits statistical significance and explains/calculates 40.2% of the variability in the pain outcome as a dependent variable (*p* < 0.001, r^2^: 40.2). The multivariate logistic regression analysis revealed that mild foraminal stenosis (OR: 3.460, 95% CI: 1.436 to 8.333, *p* = 0.006) was associated with a positive outcome. No history of previous lumbar surgery was significantly associated with a good prognosis following the PEA procedure (OR: 2.242, 95% CI: 1.067 to 4.716, *p* = 0.033). A significant correlation was present between no spondylolisthesis and a higher proportion of successful outcomes (OR: 2.976, 95% CI: 1.246 to 7.092, *p* = 0.014). Patients who had no depression obtained better treatment outcomes (OR: 3.105, 95% CI: 1.127 to 8.547, *p* = 0.028). Despite showing a trend towards statistical significance (*p* < 0.2) in univariate analysis, opioid use (OR: 0.561, 95% CI: 0.269 to 1.170, *p* = 0.123) and higher baseline VAS scores (OR: 0.787, 95% CI: 0.583 to 1.064, *p* = 0.120) were not statistically significant when subjected to the scrutiny of multivariate logistic regression (Table 4).

## 4. Discussion

The results of our study confirm the benefits of the PEA procedure in the treatment of patients with chronic lumbar radicular pain recalcitrant to conservative treatment modalities, including epidural steroid injections. Overall, our study demonstrated that PEA procedures provide an improvement of 50% or greater in terms of VAS score in 56% of patients at 12 months. Despite the widespread utilization of PEA in clinical practice and the presence of studies demonstrating its impact on pain scores, the literature is lacking in terms of comprehensive investigations into the clinical, radiological, and demographic factors that influence the efficacy of the PEA procedure. What is striking about this study is that a wide variety of characteristics that were not included in previous studies and different causes of pain gathered under the umbrella of chronic lumbar radicular pain were evaluated to predict the outcomes of PEA. In multivariate logistic regression analysis, depression, severe foraminal stenosis, previous lumbar surgery, and spondylolisthesis were associated with poor outcomes following the PEA procedure. 

Recently, PEA has become an important part of the minimally invasive procedures in the treatment of lumbar radicular pain refractory to conventional treatment modalities such as interlaminar or transforaminal epidural steroid injections [11,12,13]. The PEA procedure involves mechanical adhesiolysis, manipulating the catheter tip to reach the nerve root and epidural space, breaking up the scar formation, and allowing the delivery of high concentrations of medications. Additionally, using hyaluronidase as a pharmacological adhesiolysis contributes to the beneficial effect of adhesiolysis. Hyaluronidase is an anti-adhesive agent used for the acceleration of the adhesiolysis of fibrous tissues and potential adhesions in the epidural and perineural space to facilitate the spread of drugs and enhance the effects of medications. It also plays a role in breaking up proteins that form proteoglycans. Performing chemical adhesiolysis by injection of hyaluronidase in addition to mechanical adhesiolysis has been investigated to improve the beneficial effect of the PEA procedure [25,26]. Due to the routine utilization of hyaluronidase in our practice, we have been unable to investigate the potential beneficial impacts of this procedure.

One of the prominent factors significantly associated with negative treatment outcomes was severe foraminal stenosis on the MRI findings. Similar to our results, several studies evaluating the outcome after PEA have found that severe foraminal stenosis is a predictor of poor prognosis [13,27,28]. Despite the presence of severe foraminal stenosis, 35% of patients included in this study reported successful treatment outcomes. Unlike the findings in this current study regarding foraminal stenosis, no statistically significant association was present between the treatment success for PEA and the grade of central canal stenosis. These findings can be explained by the central canal being relatively larger than the neural foramen, which enables the clinicians to manipulate the catheter to the target site and deliver the medication to the target lesion. While stenosis may narrow the central canal, it still tends to offer sufficient space for the advancement and placement of a catheter at the intended target sites. However, even a relatively modest reduction of around one-third in the normal diameter of the neural foramen can significantly obstruct the advancement of the catheter. For the treatment of patients with severe foraminal stenosis, using a transforaminal approach to PEA may have the potential to provide a better treatment outcome. In a prospective study, Park and Lee investigated the relationship between the degree of severity of lumbar foraminal stenosis and the effectiveness of PEA with a transforaminal approach. They found no correlation between therapeutic response and the degree of foraminal stenosis [29]. In this current study, since all PEA procedures are performed by utilizing the caudal epidural approach, the effectiveness of the caudal epidural and transforaminal approaches has not been compared with each other.

In the univariate logistic regression analysis, but not in the multivariate model, our results showed that a higher pretreatment pain score and opioid use were correlated with a poor prognosis. Several trials investigating characteristics associated with treatment results have reported that subjects with higher pain scores experienced unsuccessful treatment outcomes [30,31]. Contrarily, there have been studies that have yielded opposite results; the reasons underlying this topic remain unclear, and the debate surrounding the positive or negative impacts of higher baseline pain scores on analgesic therapies continues [32]. On the other hand, recent research investigated opioid use and found correlations between opioid use and treatment failure for interventions such as radiofrequency procedures and epidural steroid injections [30,33]. Negative treatment outcomes associated with opioid use could be explained by the development of opioid-induced hyperalgesia, secondary gain, or a lower pain threshold, which could predispose patients to treatment failure. Considering the insignificance of these results on the response to PEA in the multivariate model, coupled with the existence of contradictory findings in other studies, we maintain that these variables do not yield conclusive information. 

According to our results, the history of lumbar surgery and spondylolisthesis was associated with a negative treatment outcome in logistic regression analysis. Similar to our results, previous studies evaluating the prognostic predictors of PEA procedures reported that previous surgery and spondylolisthesis had a poor influence on the effectiveness of PEA procedures [13,34]. A main reason for this finding is the perineural fibrosis and adhesions developed after surgery that impede the catheter from accessing the target site and disrupts spread of injectate effectively. However, it must also be emphasized that this result does not suggest that PEA is not an effective treatment modality for patients with a history of surgery. Of the 58 patients who had a history of lumbar surgery, 23 (40%) had experienced clinical benefits in the treatment of pain at 12 months. Moreover, the literature regarding the clinical benefits of PEA in patients with post-lumbar surgery shows evidence that this technique is more effective than epidural steroid injections [35,36]. Spondylolisthesis not only results in segmental instability but also leads to a reduction in the cross-sectional area of the vertebral canal. Additionally, it can cause noticeable thickening and buckling of the ligamentum flavum, as well as hypertrophy of the nearby facet joints [37]. The presence of these specific structural characteristics in spondylolisthesis can cause barriers that impede the advancement of a catheter and effective adhesiolysis during PEA procedures, resulting in patients with spondylolisthesis tending to experience poorer treatment outcomes compared to those without these conditions.

One of the factors that affects the treatment success of PEA is depression. This trial’s results indicated a diminished likelihood of a positive response among patients with depression. Consistent with these findings, several studies have demonstrated that depression is associated with a poor outcome in the efficacy of interventional procedures for managing chronic pain [30,32,33]. Pain comorbid with depression is frequent, with each condition intensifying the other and exhibiting overlapping symptoms, resulting in lower treatment outcomes and limited treatment options [38]. It is important to note that pain differs from nociception and encompasses not only A delta and C fiber activation but also intricate interplays involving biological, psychological, and social elements [39]. This distinction could partly explain the correlation between depression and treatment outcomes and why interventions that address pathology often do not provide successful treatment outcomes. Although the data of this study do not support the avoidance of interventional procedures in patients with depression, the intertwined nature of pain and depression necessitates a comprehensive and multimodal approach to address both aspects simultaneously, aiming to improve quality of life and enhance the effectiveness of interventional procedures.

There were several limitations that should be noted. Firstly, as our regression analyses were focused on assessing the influence of predictive factors on outcomes at the 12th month, the ability to predict successful outcomes over the longer period remains uncertain. Secondly, the success of the treatment was confined solely to changes in pain relief. Due to the lack of routine recording in the clinic, we were unable to track changes in pain medication consumption or functional disability. Thirdly, as with many retrospective studies, there were patients who had to be excluded due to missing data. Lastly, it is important to note that this study was conducted retrospectively, lacking a control or sham group for a comparative analysis of the procedure’s outcomes. The prevalence of placebo and nocebo effects in the context of interventional treatments is estimated to range between 13% and 30% and 3% and 8%, respectively [40]. Within the scope of this study, it is noteworthy that all enrolled patients were refractory to conventional therapeutic modalities as well as repeated epidural steroid injections. Taking into account these observations and the inherently progressive and degenerative nature of refractory chronic lumbar back pain, we assert that the pain experienced by our patients has reached a plateau. The observed pain relief following the intervention is attributed to PEA rather than being a consequence of the natural resolution of lumbar radicular pain or a placebo effect. To overcome this constraint, a randomized controlled trial may be necessary, offering a more comprehensive approach to address this limitation.

## 5. Conclusions

This study suggests that the PEA procedure is an effective treatment option for pain relief in patients with lumbar radicular pain who are refractory to conservative treatments, including epidural injection. Previous lumbar surgery, depression, spondylolisthesis, and severe foraminal stenosis were found to be significant predictors of the poor prognosis of PEA. Refining patient selection based on the findings of this study has the potential to optimize the balance between benefits and risks and improve treatment efficacy. Prospective randomized controlled trials are needed to definitively establish the validity of these factors.

## Figures and Tables

**Table 1:** Demographics of the study population.

Variable	*n* = 193
Age (yr)	61.9 ± 10.4
Sex Female Male	- 97 (50.2%) 96 (49.8%)
History of smoking Current Former Never	- 28 (14.5%) 76 (39.3%) 89 (46.1%)
Working status Employed Unemployed (retired, housewives, etc)	- 74 (38.3%) 119 (61.6%)
Obesity Present Absent	- 65 (33.7%) 128 (66.3%)
History of depression Yes No	- 39 (20.2%) 154 (79.8%)
History of spondylolisthesis Yes No	- 45 (23.3%) 148 (76.7%)
History of lumbar surgery Yes No	- 58 (30%) 135 (70%)
Use of opioid Yes No	- 72 (37.3%) 121 (62.7%)
Duration of pain (months)	10.1 ± 3.72
Baseline VAS score	7.06 ± 1.30

The data were presented as a mean ± SD or absolute number (percentage). VAS: visual analogue scale.

**Table 2:** Univariate logistic regression of the demographic and baseline clinical factors associated with treatment outcomes for percutaneous epidural adhesiolysis.

	Positive Outcome (*n* = 109)	Negative Outcome (*n* = 84)	Odds Ratio (95% CI)	*p*
Age	62.3 ± 9.2	61.3 ± 10.2	1.013 (0.97, 1.05)	0.561
Sex Female Male	- 57 (52.3%) 52 (47.7%)	- 40 (52.3%) 44 (52.4%)	- 1.026 (0.47, 2.2) 1 (Ref)	0.947
Smoking status Current Former Never	- 12 (11%) 46 (42.2%) 51 (46.8%)	- 16 (19%) 30 (35.7%) 38 (45.3%)	- 1 (Ref) 1.548 (0.51, 4.64) 1.844 (0.61, 5.51)	- - 0.436 0.273
Working status Employed Unemployed (re-tired, housewives, etc.)	- 41 (37.6%) 68 (62.4%)	- 33 (39.3%) 51 (60.7%)	- 1 (Ref) 1.116 (0.44, 2.80)	- - 0.815
Obesity Present Absent	- 36 (33%) 73 (67%)	- 29 (34.5%) 55 (65.5%)	- 1.165 (0.53, 2.56) 1 (Ref)	0.702
Depression Yes No	- 8 (7.3%) 101 (92.7%)	- 31 (36.9%) 53 (63.1%)	- 1 (Ref) 2.750 (0.95, 8.33)	- - 0.062
Spondylolisthesis Yes No	- 13 (11.9%) 96 (88.1%)	- 32 (38.1%) 52 (61.9%)	- 1 (Ref) 3.831 (1.40, 11.11)	- - 0.009
History of lumbar surgery Yes No	- - 23 (21.1%) 86 (78.9%)	- - 35(41.7%) 49 (58.3%)	- - 1 (Ref) 2.257 (1.03, 5)	- - - 0.043
Use of opioid Yes No	- 35 (32.1%) 74 (67.9%)	- 37 (44%) 47 (56%)	1 (Ref) 2.090 (0.961, 4.76)	- - 0.064
Duration of pain (months)	9.87 ± 3.16	10.3 ± 4.34	0.985 (0.88, 1.09)	0.787
Grade of central stenosis Mild Moderate Severe	- 50 (46.7%) 43 (40.2%) 14(13.1%)	- 35 (44.3%) 31 (39.2%) 13 (16.5%)	- 1.209 (0.40, 3.57) 1.077 (0.47, 2.5) 1 (Ref)	- 0.759 0.859
Grade of foraminal stenosis Mild Moderate Severe	- 55 (51.4%) 32 (29.9%) 20 (18.7%)	- 18 (22.5%) 26 (32.5%) 36 (45%)	- 4.670 (1.81, 12.50) 2.182 (0.90, 5.55) 1 (Ref)	- 0.002 0.085
Baseline VAS score	6.75 ± 1.17	7.50 ± 1.28	0.626 (0.59, 4.14)	0.127

Positive outcome was described as a 50% or more reduction in the visual analog scale lasting at least 12 months. Negative outcome defines <50% pain relief or not lasting for 12 months. Data are expressed as numbers (%) and mean standard deviation. *p* value compares positive outcome vs. negative outcome. *p* values were italicized and written in bold to represent statistical significance. CI: confidence intervals, VAS: visual analogue scale.

**Table 3:** Univariate logistic regression of the procedural factors associated with treatment outcomes for percutaneous epidural adhesiolysis.

	Positive Outcome (*n* = 109)	Negative Outcome (*n* = 84)	Odds Ratio (95% CI)	*p*
Target level 1 level 2 levels 3 levels	- 35 (32.1%) 62 (56.8%) 12 (11.0%)	- 25 (29.7%) 48 (57.1%) 11 (13.0%)	- 1.398 (0.520–3.759) 1.082 (0.538–2.164) Ref	- 0.506 0.824
Target side Left Right Both Central Left, central Right, central Both, central	- 12 (11.0%) 15 (13.7%) 30 (27.5%) 4 (3.6%) 10 (9.1%) 14 (12.8%) 24 (22.0%)	- 10 (11.5%) 7 (8.3%) 23 (27.3%) 4 (4.7%) 11 (13.0%) 11 (13.0%) 18 (21.4%)	- Ref 1.832 (0.525–6.402) 1.111 (0.405–3.053) 0.840 (0.163–4.328) 0.765 (0.228–2.575) 1.065 (0.329–3.454) 1.160 (0.401–3.359)	- - 0.343 0.838 0.834 0.666 0.916 0.784
Number of target side 1–2 3–4 >4	- 30 (27.5%) 56 (51.3%) 23 (21.1%)	- 25(29.7%) 44 (52.2%) 15 (17.8%)	- Ref 1.020 (0.519–2.003) 1.274 (0.527–3.080	- - 0.954 0.591

Positive outcome was described as a 50% or more reduction in the visual analog scale lasting at least 12 months. Negative outcome was defined as < 50% pain relief or not lasting for 12 months. Data are expressed numbers (%). CI: confidence intervals.

**Table 4:** Multivariate logistic regression of the factors associated with positive outcomes for percutaneous epidural adhesiolysis.

Predictor	Odds Ratio	95% Confidence Interval	*p*
Grade of foraminal stenosis Mild Moderate Severe	- 3.460 1.890 1 (Ref)	1.436, 8.333 0.843, 4.329	- 0.006 0.121
Use of opioid Yes No	- 1 (Ref) 1.782	- - 0.854, 3.717	- - 0.123
Baseline VAS score	0.787	0.583, 1.064	0.120
History of spondylolisthesis Yes No	- 1 (Ref) 2.976	- - 1.246, 7.092	- - 0.014
History of lumbar surgery Yes No	- 1 (Ref) 2.242	- - 1.067, 4.716	- - 0.033
History of depression Yes No	- 1 (Ref) 3.105	- - 1.127, 8.547	- - 0.028

VAS: Visual analog scale.

## Data Availability

Present with the corresponding author and can be delivered upon reasonable request.
